# Spatial–Temporal Transformer Networks for Traffic Flow Forecasting Using a Pre-Trained Language Model

**DOI:** 10.3390/s24175502

**Published:** 2024-08-25

**Authors:** Ju Ma, Juan Zhao, Yao Hou

**Affiliations:** School of Mechanical Engineering and Electronic Information, China University of Geosciences, Wuhan 430074, China

**Keywords:** traffic flow forecasting, spatial–temporal dependency, Transformer, LLMs

## Abstract

Most current methods use spatial–temporal graph neural networks (STGNNs) to analyze complex spatial–temporal information from traffic data collected from hundreds of sensors. STGNNs combine graph neural networks (GNNs) and sequence models to create hybrid structures that allow for the two networks to collaborate. However, this collaboration has made the model increasingly complex. This study proposes a framework that relies solely on original Transformer architecture and carefully designs embeddings to efficiently extract spatial–temporal dependencies in traffic flow. Additionally, we used pre-trained language models to enhance forecasting performance. We compared our new framework with current state-of-the-art STGNNs and Transformer-based models using four real-world traffic datasets: PEMS04, PEMS08, METR-LA, and PEMS-BAY. The experimental results demonstrate that our framework outperforms the other models in most metrics.

## 1. Introduction

An efficient transportation system significantly reduces traffic congestion, enhances transportation efficiency, lowers logistics costs, and promotes economic growth. Intelligent transportation systems (ITSs) are crucial in modern city management and transportation planning. By collecting and analyzing traffic data, ITSs provide a scientific foundation for urban planning and transportation management. Additionally, ITSs can enhance infrastructure safety through traffic load modeling [1,2].

Traffic flow forecasting is a crucial part of ITS and a significant branch of spatial–temporal prediction. It involves analyzing historical traffic conditions, modeling the spatial–temporal dependencies in traffic flows, and using these data to estimate future traffic conditions at a specific location. The complexity of modeling spatial–temporal dependencies challenges traffic forecasting.

Classical statistical methods such as autoregressive integrated moving average (ARIMA) and seasonal autoregressive integrated moving average (SARIMA) have been used to solve short-term traffic flow forecasting problems [3,4,5]. Statistical models are interpretable. However, statistical methods require predefined structures and usually assume that the data are smooth. This limits their ability to deal with the complexity and nonlinearity inherent in traffic data.

Machine learning methods do not rely on predefined structures and can automatically detect patterns in data, which are more suitable for traffic flow prediction tasks. For example, support vector machine (SVM) is an excellent algorithm for traditional machine learning. SVM-based models have good generalization properties and are relatively easy to optimize [6,7,8]. However, the hyper-parameter tuning process increases computational complexity and time. In contrast, ensemble learning methods that utilize boosting and bagging techniques not only reduce the need for extensive hyperparameter tuning, but are also easier to apply [9,10,11]. Despite this, they still face the inherent limitations of traditional machine learning models, resulting in constrained prediction accuracy.

Recently, the rapid development of deep learning has led to significant advancements in traffic flow forecasting, markedly improving prediction accuracy. Various deep learning methods, such as recurrent neural networks (RNNs) [12,13,14], temporal convolutional networks (TCNs) [15], and Transformers [16], can capture the temporal dependence in time sequences. Unlike RNNs and TCNs, Transformer models sequence data entirely on an attention mechanism, enabling efficient parallel computation. Transformer has been recognized as a robust neural network for modeling long time sequences and has been applied to various time series tasks. Transformer’s success originated from machine translation, which translates source sentences (source sequences) from one language into target sentences (target sequences) in another language. The source and target sequences are represented as tokens before being sent into the sequence-to-sequence (Seq2seq) models [17]. Some similarities are identified between the machine translation process and traffic flow prediction. The historical traffic flow data (source sequences) are the input and the predicted future traffic flows (target sequences) are the output. Both historical and predicted sequences of traffic flows can be represented as embeddings like tokens. This similarity lays the foundation for applying Transformer to traffic flow forecasting [18].

Traffic flow constantly changes on spatial–temporal scales, and the traffic flow data collected in a specific area are based on a set of time series and are viewed as data defined over a graph domain. Traffic sensors at intersections correspond to the graph nodes, and the connections and distances between them are like the paths on the graph. These sensors can collect parameters such as the density and speed of the vehicle flow. The connectivity and distance of the paths between the sensors correspond to the edges of the graph and their weights. The traffic condition is the graph signal of those changes over time. Since graph neural networks (GNNs) [19,20,21,22] are powerful tools for processing graph data, incorporating a GNN into a sequence model constitutes spatial–temporal graph neural networks (STGNNs) [23,24,25,26], which jointly capture spatial and temporal dependencies. It is also possible to incorporate graphical information into the Transformer to support graph structure understanding within the Transformer. This combination also belongs to one of the forms of STGNNs [27,28,29]. These hybrid network structures, which can extract complex spatial–temporal correlations, make spatial–temporal modeling networks increasingly complex. Additionally, GNNs often heavily rely on predefined graph structures or focus only on GNN learning, disregarding the suboptimal nature of a graph structure that evolves and is not optimal at the current moment.

The self-attention mechanism in the Transformer allows for the dynamic modeling of spatial dependencies and capturing the real-time traffic flow. The mechanism is equivalent to updating the graph structure during the training process. The approaches developed in previous studies [30] are based on only the Transformer to extract spatial–temporal information in traffic flow, simplifying the process compared with complex STGNNs. However, temporal and spatial information is fed into the first layer of the Transformer encoder at the same time, which confuses the spatial and temporal content before the temporal correlation is extracted independently. 

The natural language processing (NLP) field has been remarkable in recent years with the development of pre-trained large language models (LLMs). These LLMs facilitate model training for various NLP downstream tasks, expanding beyond the traditional NLP scopes. However, spatial–temporal modeling has not fully benefited from the significant advancement in LLMs. While pre-trained temporal models have been applied [31,32], the largest dataset for time series analysis is much smaller than NLP [33]. As a result, there is still a lack of sufficient data to pre-train spatial–temporal foundation models. Several studies have attempted to address this gap by applying LLMs to spatial–temporal tasks [34,35,36,37,38]. For instance, [34] pioneered using LLMs for time series analysis, the input embedding layer of the LLM was retrained to project time series data into the appropriate dimensions of LLM. Ref. [35] combines the graph attention mechanism (GAT), which specializes in capturing dependencies in graph structures, with an LLM to predict the missing values in sequences. The LLM processes time series data, embedding sequence data into a high-dimensional space. Subsequently, the GAT integrates spatial information, enhancing the overall prediction accuracy. Ref. [36] attempted to align the language and time series data, and inputs the aligned time series embedding into the LLMs and using the Prompt-as-Prefix technique in LLM fine-tuning. In [37], LLMs were introduced into traffic flow forecasting for the first time, using fusion convolution to generate spatial–temporal representation and feed this representation into LLM. A recent study introduced UrbanGPT [38], which integrates a spatial–temporal dependency encoder with an instruction fine-tuning approach to better understand the intricate relationship between time and space. To seamlessly align spatial–temporal signals with LLMs, a spatial–temporal instruction tuning paradigm has been developed. This approach enables the model to generalize effectively across various urban scenarios, even in data-scarce conditions.

This study proposes a spatial–temporal transformer network (STTLM) incorporating a pre-trained language model (LM) to forecast traffic flow. The key contributions of this study are summarized as follows:

(1) We have developed a framework to extract spatial–temporal features from traffic data using the Transformer’s self-attention mechanism and the design of embeddings to extract spatial–temporal dependencies.
(2) Our approach involves using the temporal Transformer (TT) first to extract the features related to temporal information separately. Then, these features are input into the spatial Transformer (ST) together with the unique embedding associated with spatial data. This method realizes the fusion of spatial–temporal information, avoids the confusion of spatial-temporal details during the initial self-attention process, and maximizes the role of embedding in the model.
(3) Additionally, we utilized pre-trained language models to improve sequence prediction performance without the need for complex temporal and linguistic data alignment.

## 2. Related Work

### 2.1. TT and ST

Time series data are input into the Transformer to learn temporal dependencies, like what is carried out in NLP: each time step value is considered an independent token [18]. Transformer used in this manner is known as the TT. A previous study [39] revealed the effectiveness of Transformer in graph learning, where the graph nodes were considered independent tokens. In this scenario, the attention mechanism is used to incorporate the structure at the node level and the relationships between the nodes. The Transformer used in this scenario is called the ST. Models for traffic flow forecasting have integrated TTs and STs to enhance accuracy and effectiveness [30].

### 2.2. Embedding

Utilizing an embedding layer before the backbone network to create multiple embeddings for the model is a simple but powerful technique. Ref. [18] introduced four strategies for encoding the temporal embeddings of traffic flows to capture the continuity and periodicity of traffic series, and promote temporal dependency modeling. A total of seven temporal encoding methods were generated by combining the different strategies: relative/global position encoding, relative/periodic position encoding, global/periodic position encoding, and time series segments. Furthermore, spatial embeddings have been leveraged in multivariate time series prediction models [40,41] to incorporate information such as traffic delay. These embeddings have been applied and achieved better results.

Temporal relations in traffic time series are influenced not only by periodicity but also by the order in which events occur. Different sensors can capture distinct temporal patterns, yet traffic data from neighboring locations typically show similar values. To enhance the effectiveness of embeddings, [30] proposed a new spatial–temporal adaptive embedding method, which avoided the use of predefined or dynamic adjacency matrices for modeling spatial relationships. This approach improved the model’s sensitivity to temporal order and better simulated complex traffic patterns from the original data.

### 2.3. Pre-Trained LM

The concept of pre-training is closely linked to transfer learning [42], which involves reusing knowledge acquired from source tasks or source domains and applying it to target tasks or target domains. Traditionally, transfer learning relies on labeled data for supervised training. However, deep transfer learning has shifted this paradigm towards pre-training followed by fine-tuning. Self-supervised learning on large volumes of unlabeled data has become the norm, which enables the application of pre-trained models to various downstream tasks through fine-tuning.

Pre-training in NLP usually refers to language modeling based on a corpus [43]. For example, the bidirectional encoder representations from transformers (BERT) [44] builds language models based on transformer encoders. BERT performs a masked language modeling (MLM) pre-training task similar to completing the blanks, which recovers masked tokens in the input sentence based on the context. BERT serves as the foundation for a family of LMs in various NLP tasks [45].

The generative pre-trained Transformer (GPT) [46] focuses on generative tasks in NLP. The GPT models use the Transformer decoder as the backbone, and the model performance improves as the number of layers and parameters increase. Above a certain size of parameters, LM becomes an LLM. The GPT family of LLMs has had a profound impact in a number of areas related to artificial intelligence (AI). In addition, high-quality open-source LLMs that can be deployed privately, such as Mistral [47] and Llama [48], have also been widely adopted, contributing even more to the development of AI.

### 2.4. LLM Fine-Tuning

Fine-tuning the LLMs has become an important technique, which improves the effectiveness of the model in specific tasks [49,50]. There are two approaches to fine-tuning an LLM: full parametric fine-tuning (FFT) and parameter-efficient fine-tuning (PEFT) [51,52].

FFT is a widely adopted traditional method. This method retrains all the parameters of a pre-trained model and thus consumes a lot of resources. With sufficient computational resources, FFT provides the best model performance and task adaptability. Ref. [53] proposed optimization strategies for FFT, such as distributed training, parameter sharing, and mixed-accuracy training, to improve model performance while reducing computational overhead. 

Low-rank adaptation (LoRA) [54] is a representative PEFT technique. It significantly reduces the number of model parameters that need to be updated. It decomposes the weight matrices of a pre-trained model into low-rank matrices and trains only these decomposed matrices. This approach not only reduces the training cost while maintaining model performance, but also enhances the flexibility and applicability of fine-tuning.

Prompt tuning [55] can also be categorized as PEFT. Broadly speaking, prompt tuning includes in-context learning [56], instruction-tuning [57] and chain-of-thought [58]. It optimizes model performance by adjusting the model’s responses to specific instructions or tasks. This kind of method is advantageous in resource-constrained environments and can be combined with other fine-tuning methods, such as FFT and LoRA, to further enhance model performance. 

Each of these methods has distinct advantages. Understanding these methods’ strengths and limitations allows for the strategic application of fine-tuning techniques to optimize LLMs for various use cases.

## 3. Method

### 3.1. Embedding Layer

We used $\left[t-\left(T-1\right),\;t-\left(T-2\right),\ldots ,t\right]$ to represent the time steps of the historical (source) traffic series and [t+1,t+2,…,t+T] to forecast (target) traffic series. The source sequence is denoted by $\chi_{t}^{t-(T-1)}$, and the target sequence is denoted by $\chi_{t+1}^{t+T}$. $\chi_{t+1}^{t+T}$ consists of a matrix of traffic features from time step t+1 to t+T, formulated as [$X_{t+1}$, $X_{t+2}$, …, $X_{t+T}$], where each $X_{i}$∈$R^{N\times dₛ}$, and $\chi_{t}^{t-(T-1)}$, and $\chi_{t+1}^{t+T}$∈$R^{T\times N\times dₛ}$. *N* is the number of nodes, and dₛ is the dimension of the input features, equal to 1. The detailed process of generating embeddings based on source sequences is described as follows:

- $E_{k}$: To maintain the native information in the source sequences, we put $\chi_{t}^{t-(T-1)}$ through a fully connected layer of $d_{s}\times d_{1}$ to obtain the feature embedding $E_{k}$, $E_{k}\in R^{T\times N\times d_{1}}$.
- $E_{ep}$: Positional encoding is incorporated when using a Transformer as a language model [14]. $E_{ep}\in R^{T\times N\times d_{2}}$. A study [18] introduced relative position coding and global position coding for the temporal continuity of traffic flow. Both methods use hard coding (e.g., predefined sine/cosine functions, and precomputed values). Temporal continuity encoding can be obtained through training and hard coding. We first assign a random value to $E_{ep}$ and let it learn the time continuity of the traffic sequence during training.
- $E_{p}$: Unlike natural language, time series contain periodicity and temporal continuity information. For instance, traffic flow, characterized simultaneously on different days, may be extremely similar, and the same embedding can be designed for data in the traffic sequence at the same moment. Similarly, the same day in different weeks can correspond to the same embedding [30]. $E_{w}$ denotes the embedding of the weekly cycle, and $E_{d}$ denotes the embedding of the daily cycle. The weights of the embedding layer are also randomly assigned first and then trained. $E_{w},E_{d}\in R^{T\times N\times d_{3}}$, $E_{p}$=$E_{d}\left\|E_{w}\right.$, $E_{p}\in R^{T\times N\times {2d}_{3}}$.
- $E_{s}$: Time series from two traffic nodes that are physically close to each other may have a time difference even if their waveforms are similar. The embeddings mentioned above do not reflect this association. However, if the time domain waveforms are transformed into another transform domain, the effect of the time difference can be removed, showing a strong spatial node correlation. $E_{s}$ is viewed as an embedding generated based on the information in the transform domain to represent spatial information obtained based on DFT or wavelet transform. As a result, we use Harr wavelet to perform wavelet transform on the traffic series of each node. The coefficients *l* of the low-pass filter and coefficients *h* of the high-pass filter in the Harr wavelet transform are calculated as follows:
  (1) $$l[n]=\left\{\begin{array}{l} \frac{1}{\sqrt{2}}\;\;\;\;n=0\\ \frac{1}{\sqrt{2}}\;\;\;\;n=1\\ 0\;\;\;\;otherwise \end{array}\right.\;\;\;\;\;\;\;h[n]=\left\{\begin{array}{l} \frac{1}{\sqrt{2}}\;\;\;\;n=0\\ -\frac{1}{\sqrt{2}}\;\;\;\;n=1\\ 0\;\;\;\;otherwise \end{array}\right.$$
- The approximate coefficients $x_{A}$ and detail coefficients $x_{D}$ are calculated as follows:
  (2) $$\begin{array}{l} x_{A}[k]=\sum_{n}x[n]\;l[k-n]\\ x_{D}[k]=\sum_{n}x[n]\;h[k-n]\;\;\;k\in 0,1,\ldots ,T-1 \end{array}$$
- Then, the spatial embedding $E_{s}=x_{A}\left\|x_{D}\right.,E_{s}\in R^{T\times N\times d_{4}}$.

We take $d_{1}$, $d_{2}$, $d_{3}$, and $d_{4}$ to be of the same length $d_{h}$, the spatial embedding $E_{s}\in R^{T\times N\times d_{h}}$, and the temporal embedding $E_{t}=E_{k}\left\|E_{ep}\right.\left\|E_{p}\right.$, $E_{t}\in R^{T\times N\times {4d}_{h}}$.

### 3.2. Network Structure

**Figure 1:**
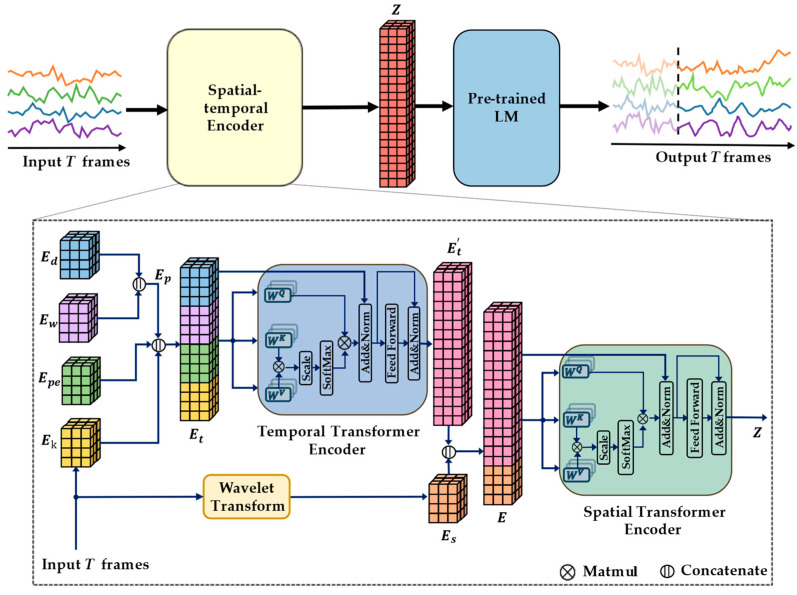
Framework of STTLM.

Figure 1 demonstrates the STTLM framework, which consists of a spatial–temporal encoder and a pre-trained LM.

The spatial–temporal encoder consists of two components: the TT encoder and the ST encoder. The TT encoder processes the temporal dependency first, while the ST encoder handles the spatial dependency and integrates the spatial–temporal information.

Each time step’s embeddings from the temporal embedding are input into the TT encoder. The output $Z^{\prime }$ of the self-attention layer is computed using the Scaled Dot-Product Attention mechanism.

(3) $$A^{\left(t\right)}=Soft\max(\frac{(E_{t}W_{Q}^{\left(t\right)}){\left(E_{t}W_{K}^{\left(t\right)}\right)}^{T}}{\sqrt{d_{h}}})$$

(4) $$Z^{\prime }=A^{\left(t\right)}(E_{t}W_{V}^{\left(t\right)})$$

where $W_{Q}^{\left(t\right)}$, $W_{K}^{\left(t\right)}$, and $W_{V}^{\left(t\right)}\in R^{{4d}_{h}\times 4d_{h}}$ are the learnable weight matrix. $A^{\left(t\right)}\in R^{N\times T\times T}$, is the attention score matrix that captures the temporal dependencies within the respective time series of the *N* nodes. Then, $Z^{\prime }$ goes through layer normalization, skip connection, and FFN layers to finally obtain the output of the TT $E_{t}^{\prime }$, $E_{t}^{\prime }\in R^{T\times N\times {4d}_{h}}$.

**Figure 2:**
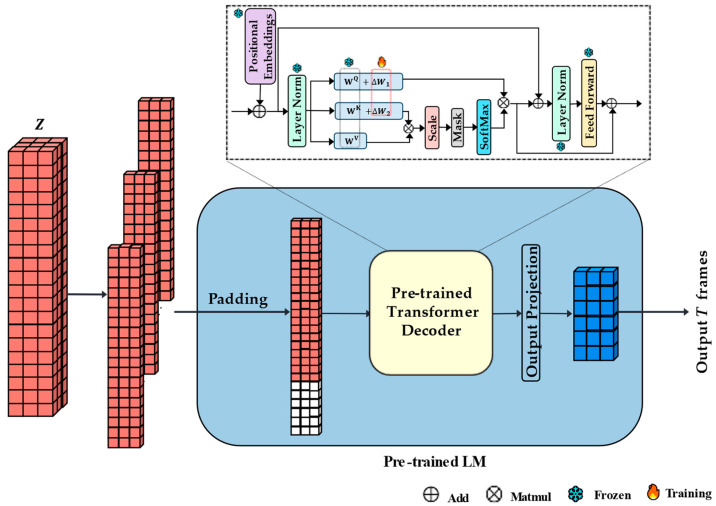
Pre-trained LM in STTLM.

As shown in Figure 2, we expand Z into $\left[Z_{1},Z_{2},\ldots ,Z_{N}\right]$ by the number of nodes before inputting Z into a pre-trained LM, $Z_{i}\in R^{T\times 5d_{h}}$. The embedding of each time step of $Z_{i}$ is put into LM as a token. Here, 5$d_{h}$ must be extended through padding to fit the input length $d_{x}$ of the hidden layer in LM. The output of the last hidden layer in LM is projected into target sequences $\chi_{t+1}^{t+T}$ through a linear layer.

## 4. Experiment Details

### 4.1. Dataset

We evaluated the algorithms’ performance on four real-world traffic prediction datasets: PEMS04, PEMS08, PEMS-BAY, and METR-LA. The METR-LA traffic dataset contains traffic information collected from 207 Los Angeles Freeway Loop sensors. The PEMS-BAY, PEMS04, and PEMS08 traffic datasets were collected by the California Department of Transportation (CalTrans) Performance Measurement System (PeMS) [59]. The PEMS-BAY traffic dataset contains traffic information collected from 325 sensors in the Bay Area. The sampling interval for each dataset was 5 min, and the details are shown in Table 1.

### 4.2. Implementation

METR-LA and PEMS-BAY are divided into training, validation, and test sets in a ratio of 7:1:2. In contrast, PEMS04 and PEMS08 are divided in a ratio of 6:2:2 ratio. Training and test sets were obtained sequentially. If the history–prediction data pairs are shuffled before being divided according to the ratio, the prediction performance will be significantly improved. For example, the 1 h mean absolute error (MAE) based on the METR-LA dataset can be reduced to 3.00, which is much better than the results 3.31 shown in Table 2. However, to be consistent with the baselines used for comparison, the test dataset is still taken from the last 20% part of the sequence

The proposed model was implemented with Pytorch 2.0.1 on an NVIDIA RTX 3090 GPU (NVIDIA, Santa Clara, CA, USA). The temporal Transformer encoder was set up with three layers, and the spatial Transformer Encoder was set up with four layers, both with a multi-head number of 4. We employed Llama-7B as the pre-trained LM; only the parameters of one hidden layer (decoder) were used and fine-tuned using LoRA.

When performing LoRA fine-tuning, the parameters of the pre-trained LM were frozen, and only the parameters of the newly added low-rank matrices were trained. This approach significantly reduced the number of trainable parameters and lowered the GPU requirements. Assuming that the original pre-trained parameter matrix is W_0_, LoRA does not train W_0_ directly. Instead, it adds ∆W = B∙A to the frozen W_0_, where A and B are both low-rank matrices. The parameter matrix of LM becomes W with ∆W = W − W_0_. Suppose W_0_ has dimensions d × k, while A has dimensions d × r and B has dimensions r × k. Rank r is much smaller than d and k. We implemented LoRA fine-tuning by inserting the A and B of ∆W in the form of residual connections in the self-attention part (Figure 2), with r set to 32.

Table 2 provides the parameters of the models with different numbers of hidden layers used. STTLM_2L differs from STTLM by using two hidden layers from Llama-7B. As shown in Table 2, LoRA fine-tuning greatly reduced the number of trainable parameters in the pre-trained LM.

### 4.3. Metrics

We used three commonly used traffic prediction metrics [23]: MAE, root mean squared error (RMSE), and mean absolute percentage error (MAPE). Let $y=y_{1},y_{2},\ldots ,y_{M}$ denote the true values to be predicted; $\hat{y}={\hat{y}}_{1},{\hat{y}}_{2},\ldots ,{\hat{y}}_{M}$ denote the predicted value; and M denotes the number of observed samples. Then, the metrics are defined as follows:

(5) $$MAE=\frac{1}{M}\sum_{i=1}^{M}\left|{\hat{y}}_{i}-y_{i}\right|$$

(6) $$RMSE=\sqrt{\frac{1}{M}\sum_{i=1}^{M}{\left|{\hat{y}}_{i}-y_{i}\right|}^{2}}$$

(7) $$MAPE=\frac{1}{M}\sum_{i=1}^{M}\frac{\left|{\hat{y}}_{i}-y_{i}\right|}{y_{i}}$$

Based on previous work, we compared the performance of the METR-LA and PEMS-BAY datasets on horizons 3, 6, and 12 (15, 30, and 60 min). We selected the average performance of all predicted 12 horizons to evaluate the PEMS04 and PEMS08 datasets.

### 4.4. Baselines

Our proposed method was compared with several widely used baselines. Five STGNN models, DCRNN [23], GWNet [24], AGCRN [25], MTGNN [26], and the Transformer-based STAEformer [29] model, were considered. The spatial–temporal coding results from our method were also input into the four-layer (ST_4L) and seven-layer (ST_7L) Transformer decoders to compare the performance with that of pre-trained LM. The baseline methods are summarized as follows:

DCRNN: a diffusion convolutional recurrent neural network combines diffusion map convolution with RNNs.

GWNet: a spatial–temporal graph convolutional network (STGCN) that integrates diffusion graph convolution with one-dimensional unfolding graph convolution.

AGCRN: an adaptive graph convolutional recurrent neural network merges adaptive graph learning with recurrent neural networks.

MTGNN: a spatial–temporal graph convolutional network that blends graph convolution with time domain convolution.

STAEformer: a Transformer network combines spatial–temporal adaptive embedding with a Transformer encoder.

## 5. Result and Discussion

### 5.1. Main Results

As shown in Table 3 and Table 4, the red font identifies the best performance, and the bold black font identifies the second-best performance.

Our approach achieved better performance than the other models on the vast majority of the metrics. STTLM and STAEformer outperformed STGNNs to a large extent, suggesting that the transformer-based model can better capture complex spatial–temporal relationships. STTLM achieved better results than STAEformer and models using multi-layer Transformer decoders. This result shows that pre-trained LMs can improve prediction performance, although they are not trained on time series data.

### 5.2. Ablation Study

We performed ablation experiments on METR-LA and PEMSBAY datasets to evaluate the effectiveness of each embedding used in STTLM. We named three variants of our model as follows:

- w/o $E_{ep}$: It removes temporal continuity embedding.
- w/o $E_{p}$: It removes temporal periodicity embedding.
- w/o $E_{s}$: It removes spatial embedding (See Table 5).

All the embeddings enhance model performance. The time–continuity embedding $E_{ep}$ has the most significant effect on the prediction performance. $E_{s}$ is less influential, which may be due to the fact that we only use simple wavelet coefficients to generate the spatial embedding.

### 5.3. Case Study

**Figure 3:**
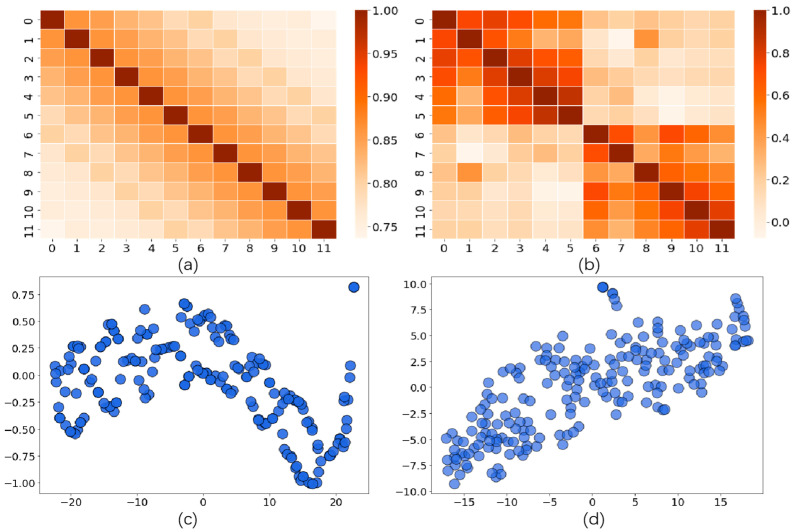
Visualization of spatial–temporal embeddings on MATR-LA (**a**) Temporal axis of $E_{t}$ (**b**) Temporal axis of Z (**c**) Spatial axis of $E_{s}$ (**d**) Spatial axis of Z.

To better understand why STTLM performs best, we took the MATR-LA dataset as an example. We visualized Embedding $E_{t}$ and Z on the temporal axis, and Embedding $E_{s}$ and Z on the spatial axis. For the temporal axis, we computed the correlation coefficient across the 12 input frames and plot heatmaps, as shown in Figure 3a,b. As can be seen from the plots, each frame is highly correlated with nearby frames, and the correlation decreased for further frames. Embedding $E_{t}$ accurately captured the temporal information in the time series (Figure 3a), and this accuracy persisted even after incorporating spatial information (Figure 3b). On the spatial axis, we used t-SNE to obtain Figure 3c,d. Figure 3c shows that the embeddings $E_{s}$ of different nodes form into clusters, which matches the spatial characteristics of the traffic data.

**Figure 4:**
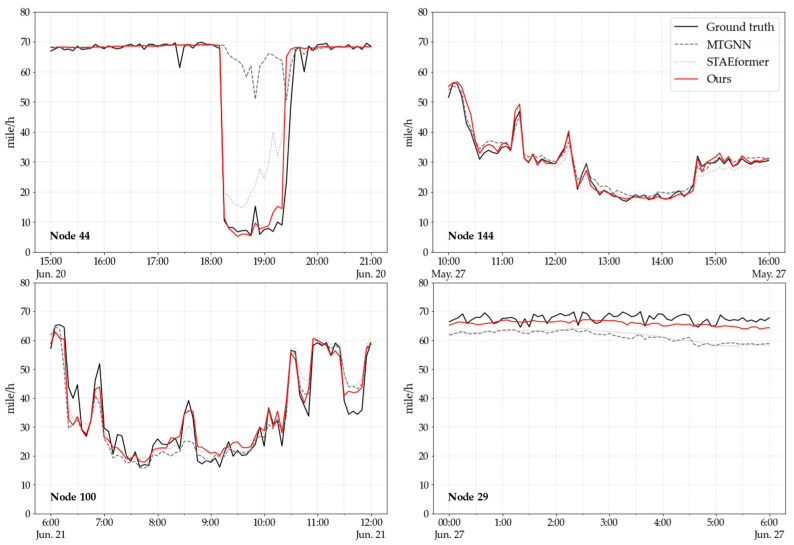
Traffic speed prediction on the METR-LA dataset.

Figure 4 further provides visualizations of the predictions of our STTLM model, the MTGNN model and STAEformer model for the dataset METR-LA. As can be observe in Figure 4, for the data captured by the four different sensors, the STTLM better match the ground truth, both in dealing with relatively stable traffic conditions and in capturing sudden changes in the traffic, followed by STAEformer, which also has a more prominent result.

### 5.4. Limitations

While LoRA fine-tuning significantly reduces the number of trainable parameters (Table 2), all parameters of the hidden layers still need to be loaded during the inference stage. This imposes high demands on GPU and limits the practical application of the model.

As mentioned in Section 5.1, using the pre-trained LM as a decoder for spatial–temporal information enhances prediction performance. However, increasing the number of hidden layers in the pre-trained LM does not necessarily lead to further performance improvements (Table 6). This might be due to the fact that, as the size of the language model grows, effective fine-tuning requires more extensive data.

## 6. Conclusions

In this study, for the proposed model STTLM, we introduced a new spatial–temporal encoder framework that facilitates spatial–temporal feature encoding for traffic data. The incorporation of a pre-trained language model is also used to improve the performance. We have successfully used this framework for traffic flow forecasting. It had excellent performance in evaluation indexes such as MAE, RMSE, and MAPE, and the forecasting results. Experiments conducted on the four benchmark datasets show that our STTLM model outperforms the baselines, demonstrating the effectiveness of our proposed framework.

In this study, only simple wavelet transform was applied to generate spatial embeddings, and we will explore how to design better spatial embeddings for the architecture in future works.

## Figures and Tables

**Table 1:** Summary of Datasets.

Dataset	Number of Sensors	Samples	Time Range
METR-LA	207	34,272	1 March 2012–27 June 2012
PEMS-Bay	325	52,116	1 January 2017–30 June 2017
PEMS04	307	16,992	1 January 2018–28 February 2018
PEMS08	170	17,856	1 July 2016–31 August 2016

**Table 2:** Number of parameters in the models.

Model	Total Params	Trainable Params	Params in Embedding Layers	Trainable Params in Pre-Trained LM
STTLM	335,076,981	1,617,525	809,704	786,432
STTLM_2L	538,246,773	2,403,957	809,704	1,572,864

**Table 3:** Performance on METR-LA and PEMS-BAY.

Dataset		Metric	DCRNN	GWNet	AGCRN	MTGNN	STAEformer	ST_4L	ST_7L	STTLM
METR-LA	3	MAE	2.67	2.69	2.85	2.69	**2.65**	3.05	2.93	2.62
		RMSE	5.16	5.15	5.53	5.16	**5.11**	6.22	5.88	5.05
		MAPE	6.86%	6.99%	7.63%	6.89%	**6.85%**	8.51%	7.84%	6.72%
	6	MAE	3.12	3.08	3.20	3.05	2.97	3.11	**2.94**	2.94
		RMSE	6.27	6.20	6.52	6.13	6.00	6.46	**5.98**	5.94
		MAPE	8.42%	8.47%	9.00%	8.16%	8.13%	8.84%	**8.03%**	7.98%
	12	MAE	3.54	3.51	3.59	3.47	**3.34**	3.74	3.64	3.31
		RMSE	7.47	7.28	7.45	7.21	**7.02**	7.87	7.64	6.88
		MAPE	10.32%	9.96%	10.47%	**9.70%**	**9.70%**	10.79%	10.25%	9.54%
PEMS-BAY	3	MAE	1.31	**1.30**	1.35	1.33	1.31	1.64	1.60	1.29
		RMSE	2.76	2.73	2.88	2.8	**2.78**	3.81	3.68	**2.78**
		MAPE	2.73%	2.71%	2.91%	2.81%	2.76%	3.72%	3.62%	**2.74%**
	6	MAE	1.65	1.63	1.67	1.66	1.62	1.62	**1.59**	1.58
		RMSE	3.75	3.73	3.82	3.77	3.68	3.77	**3.66**	3.63
		MAPE	3.71%	3.73%	3.81%	3.75%	3.62%	3.67%	**3.59%**	3.55%
	12	MAE	1.97	1.99	1.94	1.95	**1.88**	2.10	2.1	1.83
		RMSE	4.60	4.60	4.5	4.5	**4.34**	4.97	4.94	4.27
		MAPE	4.68%	4.71%	4.55%	4.62%	**4.41%**	4.82%	4.81%	4.30%

**Table 4:** Performance on PEMS03 and 08.

Dataset	Metric	DCRNN	GWNet	AGCRN	MTGNN	STAEformer	ST _4L	ST_7L	STTLM
PEMS04	MAE	19.63	18.53	19.38	19.17	**18.22**	21.36	21.35	17.73
	RMSE	31.26	**29.92**	31.25	31.7	30.18	33.36	33.20	29.31
	MAPE	13.59%	12.89%	13.40%	13.37%	**11.98%**	14.34%	14.28%	11.83%
PEMS08	MAE	15.22	14.4	15.32	15.18	**13.46**	17.06	17.02	12.82
	RMSE	24.17	23.39	24.41	24.24	**23.25**	26.86	26.78	22.36
	MAPE	10.21%	9.21%	10.03%	10.20%	**8.88%**	10.91%	10.91%	8.46%

**Table 5:** Ablation study on METR-LA and PEMS-BAY.

Dataset		Metric	w/o *E*_ep_	w/o *E*_p_	w/o *E*_s_	STTLM
METR-LA	3	MAE	2.97	2.66	2.62	2.62
		RMSE	5.84	5.06	5.06	5.05
		MAPE	7.94%	6.80%	6.79%	6.72%
	6	MAE	3.53	3.02	2.95	2.94
		RMSE	7.16	6.03	6.00	5.94
		MAPE	10.12%	8.15%	8.10%	7.98%
	12	MAE	4.34	3.42	3.32	3.31
		RMSE	8.77	6.97	6.96	6.88
		MAPE	13.38%	9.65%	9.65%	9.54%
PEMS-BAY	3	MAE	1.39	1.33	1.32	1.29
		RMSE	3.00	2.81	2.83	2.78
		MAPE	2.92%	2.91%	2.92%	2.74%
	6	MAE	1.81%	1.63	1.60	1.58
		RMSE	4.15	3.72	3.71	3.63
		MAPE	4.13%	3.76%	3.76%	3.55%
	12	MAE	2.3	1.9	1.84	1.83
		RMSE	5.27	4.41	4.31	4.27
		MAPE	5.66%	4.56%	4.46%	4.30%

**Table 6:** Performance comparison of STTLM to STTLM_2L on METR-LA and PEMS-BAY.

Dataset		Metric	STTLM_2L	STTLM
METR-LA	3	MAE	2.61	2.62
		RMSE	5.01	5.05
		MAPE	6.74%	6.72%
	6	MAE	2.93	2.94
		RMSE	5.96	5.94
		MAPE	8.03%	7.98%
	12	MAE	3.30	3.31
		RMSE	6.93	6.88
		MAPE	9.56%	9.54%
PEMS-BAY	3	MAE	1.32	1.29
		RMSE	2.81	2.78
		MAPE	2.88%	2.74%
	6	MAE	1.60	1.58
		RMSE	3.67	3.63
		MAPE	3.71%	3.55%
	12	MAE	1.85	1.83
		RMSE	4.31	4.27
		MAPE	4.44%	4.30%

## Data Availability

The METR-LA and PEMS-BAY datasets are available at https://github.com/liyaguang/DCRNN (accessed on 22 August 2024). The PEMS04 and PEMS08 datasets are available at https://github.com/XDZhelheim/STAEformer (accessed on 22 August 2024).
